# Trance in a Hysterical Woman

**Published:** 1883-09-20

**Authors:** 


### TRANCE IN A HYSTERICAL WOMAN.

Rosenthal has recorded an interesting case of trance in a hysterical woman, in which a country practitioner had declared to have ensued, as a looking-glass held to the mouth did not show any moisture, and melted sealing-wax dropped on the skin caused no reflex movements. Rosenthal, who was accidentally present, found the skin pale and cold, the pupils contracted and insensible to light, the upper and lower extremities relaxed, the heart's impulse and the radial pulse imperceptible. Auscultation, however, showed a feeble, dull, and intermittent sound in the cardiac region. No respiratory murmurs were audible. All the muscles of the face and extremities responded well to the faradic current. Although the patient had been apparetly dead for 32 hours, he thereupon informed the relations that it was only a trance, and recommended that attempts at resuscitation should be perseveringly followed.

On the follwing day he received a telegram to say that the patient awoke spontaneously twelve hours afterwards, and gradually recovered. Four months afterwards, the patient called upon him, and informed him that she knew nothing of the commencement of her attack of lethargy; that she had afterwards heard the people about her talk of her, but had been utterly unable to give the slightest sign of life. Two years afterwards, she was still alive and tolerably well.—*Weekly Med. Review.*

